# Impact of free provision of disinfectant wipes combined with bundle management on the prevention of multi-drug resistant organism infections in the respiratory and intensive care unit

**DOI:** 10.3389/fcimb.2025.1581545

**Published:** 2025-07-08

**Authors:** Yu-Dan Wang, Jian-Hong Liang, Xie-Nan Gao, Li-Li Ma, Shen-Shen Huang, Yong-Mei Zhang

**Affiliations:** ^1^ Department of Respiratory and Critical Care Medicine, The First Affiliated Hospital, and College of Clinical Medicine of Henan University of Science and Technology, Luoyang, China; ^2^ Department of Infection Control, The First Affiliated Hospital, and College of Clinical Medicine of Henan University of Science and Technology, Luoyang, China; ^3^ Department of Ophthalmology, The Yiluo Hospital of Luoyang, The Teaching Hospital of Henan University of Science and Technology, Luoyang, China

**Keywords:** respiratory and intensive care unit, multi-drug resistant organisms, disinfectant wipes, bundle management measures, prevention

## Abstract

**Objective:**

To explore the role of free provision of disinfectant wipes combined with bundle management in preventing Multi-Drug Resistant Organism (MDRO) infections in the Respiratory and Intensive Care Unit (RICU).

**Methods:**

This study included patients admitted to the RICU between January 2022 and June 2023 (control group) and from July 2023 to June 2024 (intervention group), all of whom met the inclusion criteria. The control group received standard bundle management measures, while the intervention group received unlimited use of disinfectant wipes combined with bundle management. The MDRO discovery rates and hospital-acquired infection incidence rates were compared between the two groups to assess the impact of free disinfectant wipe provision on MDRO infection prevention in the RICU.

**Results:**

There were no significant differences between the two groups in terms of patient age and baseline characteristics, except for Sex and the proportion of patients with chronic respiratory diseases. Notably, the MDRO discovery rate, MDRO infection rate, and hospital-acquired infection incidence were all significantly lower in the intervention group compared to the control group (MDRO discovery rate: 35.2% vs 45.9%, P<0.001; MDRO infection rate: 0.8% vs 3.5%, *P* = 0.003; hospital-acquired infection rate: 1.5% vs 3.52%, P=0.030). However, the intervention group had more percentage of patients receiving mechanical ventilation and longer ICU stay (P < 0.05). Furthermore, in-hospital mortality was lower in the intervention group (19.5% vs 13.5%, *P* < 0.05).

**Conclusion:**

The combined intervention of unlimited use of disinfectant wipes with bundle management significantly reduced MDRO hospital-acquired infection rates and in-hospital mortality in the RICU, demonstrating its effectiveness in infection prevention.

## 1 Introduction

Multi-drug resistant organism (MDRO) infections have become a significant global public health challenge, severely threatening patient health and increasing the burden on healthcare systems. The emergence and spread of resistant bacteria significantly reduce the effectiveness of conventional antibiotics, leading to suboptimal treatment outcomes, prolonged hospital stays, and adversely affecting patient survival rates and quality of life (Wang et al., 2016; Ding et al., 2023). According to the Global Burden of Disease Database, approximately 4.71 million people worldwide died from antibiotic-resistant infections in 2021, with 1.14 million deaths attributed specifically to resistant bacterial infections. The study further predicts that by 2050, around 8.82 million deaths will occur due to antimicrobial resistance, with 1.91 million of these directly caused by resistant bacterial infections (Collaborators, 2024). Antibiotic resistance has become one of the most critical threats to global health, food safety, and economic development (Hou et al., 2023; Hassan et al., 2024). Therefore, identifying effective preventive and control measures is crucial to combat the spread of MDROs.

The intensive care unit (ICU), as a primary setting for the care of critically ill patients, is often a hotspot for MDROs and hospital-acquired infections. ICU patients are typically elderly, have multiple comorbidities, and exhibit weakened immune functions, with many receiving extensive antimicrobial treatment, making them highly susceptible to MDRO infections (Assefa, 2022). Environmental surfaces, as high-touch areas, have been shown to be significant reservoirs and transmission routes for pathogens, especially MDROs. Therefore, effective environmental cleaning and disinfection, particularly of surface areas, are essential in preventing potential infections and reducing the risk of MDROs and hospital-acquired infections. In recent years, with the spread of multidrug-resistant, extensively drug-resistant, and pandrug-resistant bacteria, environmental disinfection has faced unprecedented challenges (Wille et al., 2018; Obenhuber et al., 2024). Currently, chlorine-based disinfectants remain the most commonly used in clinical settings, but their strong corrosive properties, irritating odor, and complicated application often pose inconveniences to the disinfection process (Li et al., 2023; Wozniak et al., 2024). In contrast, disinfectant wipes, as a convenient, safe, and efficient cleaning tool, have been increasingly adopted (von Dehn et al., 2022; Albaharna et al., 2023). Studies have demonstrated that disinfectant wipes can effectively eliminate MDROs from surfaces and significantly reduce the MDRO infection rates in hospitalized patients (von Dehn et al., 2022; Dadon et al., 2023).

Although ICUs have implemented bundle management measures to reduce the risk of MDRO infections (Song et al., 2024), no studies have yet explored the effect of combining unlimited use of disinfectant wipes with bundle management. To evaluate the effectiveness of this practice, this study adopted a retrospective cohort design, systematically collecting clinical data from patients and comparing the outcomes of unlimited use of disinfectant wipes combined with routine bundle management versus bundle management alone. The study aimed to assess the differences between the two intervention strategies in terms of MDRO infection rates and hospital-acquired infection rates, providing scientific evidence for the development of hospital infection control strategies, and offering an effective intervention approach to improve clinical outcomes for patients.

## 2 Materials and methods

### 2.1 Study subjects

This study focused on 1,908 patients admitted to the Respiratory and Intensive Care Unit (RICU) of the First Affiliated Hospital of Henan University of Science and Technology between January 2022 and June 2024. The inclusion and exclusion criteria are as follows:

#### 2.1.1 Inclusion criteria

Patients who were admitted to the RICU for the first time and had a hospitalization duration of ≥ 24 hours.

#### 2.1.2 Exclusion criteria

1. Patients who were transferred in and out of the RICU multiple times during a single hospitalization.
2. Patients with confirmed MDRO infection upon admission to the RICU.
3. Patients with a hospital stay of less than 24 hours.

### 2.2 Study groups

The patients were divided into two groups: the control group (January 2022 to June 2023) and the intervention group (July 2023 to June 2024). The specific grouping is as follows:

#### 2.2.1 Control group

The control group consisted of patients admitted to the RICU between January 2022 and June 2023, who received standard bundle management measures. These measures included:

1. Active MDRO Screening: All patients transferred to the RICU underwent routine pathogen screening based on their clinical condition (e.g., throat swabs, bronchoalveolar lavage, rectal swabs, etc.) to accurately differentiate hospital-acquired infections from community-acquired infections. Positive results prompted immediate isolation in a designated area with strict contact isolation measures.
2. Isolation Measures: Based on screening results, positive patients were isolated in single rooms or placed with patients infected by the same pathogen. Isolation signs were posted at the patient’s bedside, and medical staff were required to follow strict protocols for donning and doffing protective gear to prevent cross-contamination.
3. Cleaning and Disinfection: The patient care area underwent routine cleaning and disinfection at least twice a day. A designated team regularly cleaned medical equipment with chlorine-based disinfectants twice daily, and nursing staff were responsible for spot disinfecting patient bed units. Immediate disinfection of surfaces using disinfectant wipes was performed when contamination occurred. Floor, bed, ceiling, and bedside table surfaces were cleaned twice daily with microfiber cloths (General Administration of Quality Supervision, 2012; Ministry of Health of the People’s Republic of China, 2012).
4. MDRO Critical Value Management: Upon detecting an MDRO infection, the microbiology laboratory promptly notified the department, and an automatic system alerted the physicians, ensuring timely isolation and treatment measures.
5. Antibiotic Stewardship: Strict control over the use of antibiotics was enforced to avoid misuse and reduce the development of MDROs.
6. Infection Control Nurse Supervision: Infection control nurses conducted regular compliance checks on infection prevention measures to ensure effective implementation of the strategies.
7. Environmental Disinfection and Waste Management: Patient rooms, surrounding environments, floors, and items were disinfected at least twice daily. Infectious waste, including used dressings, was handled according to specified guidelines using double-layered medical waste bags.

#### 2.2.2 Intervention group

The intervention group included patients admitted to the RICU between July 2023 and June 2024. In addition to standard bundle care, these patients received enhanced environmental disinfection measures, centered on a “one-step” cleaning protocol using disinfectant wipes (Zhuoshi Disinfectant Wipes for Surfaces; Wuhan Zhuoshi Co., Ltd., Batch No. 20230505). Each wipe was made of non-woven fabric saturated with a composite quaternary ammonium salt solution (active ingredient concentration: 2200–2800 mg/L), and each pack contained 80 wipes.

The wipes were provided free of charge and used regularly by healthcare staff to clean frequently touched surfaces and equipment. Based on ward protocols, the minimum expected usage was 34 wipes per bed per day, with each pack intended to last approximately two days. Cleaning staff disinfected surfaces such as monitors, pumps, ventilators, and bedside tables twice daily. Primary nurses used wipes after routine care and procedures (every 4 hours) and for spot cleaning as needed. Physicians cleaned instruments such as bronchoscopes, ECG machines, and ultrasound probes after use. Respiratory therapists disinfected respiratory equipment following each session.

To ensure compliance, infection control nurses monitored total and per-bed wipe usage monthly, with year-over-year and month-over-month comparisons. H1-shift nurses were responsible for daily restocking and monitoring wipe expiration and usage timelines. In addition, environmental hygiene assessments were conducted quarterly, requiring post-disinfection surface colony counts to remain ≤5 CFU/cm².

### 2.3 Clinical data collection

For all patients meeting the inclusion criteria, basic information and key clinical data were systematically collected, including but not limited to: hospitalization number, gender, age, primary diagnosis, use and duration of risk factors such as mechanical ventilation, central venous catheters, and urinary catheters, disinfection compliance rate, bacteriological specimen submission status, MDRO infection and hospital-acquired infection rates, ICU length of stay, and clinical outcomes.

### 2.4 Specimen collection and transport

To ensure specimen quality, standardized collection and transportation methods were followed, including:

1. Throat Swabs: Both sides of the tonsils and posterior pharyngeal wall were swabbed, avoiding contact with the tongue and oral mucosa. Specimens were sent to the lab within two hours.
2. Rectal Swabs: The swab was inserted into the rectum and rotated before being sent to the lab within two hours.
3. Bronchoalveolar Lavage (BAL): BAL specimens were collected through a fiberoptic bronchoscope, with the sample being aspirated into a sterile collection cup.
4. Airway Aspiration: For patients with artificial airways, specimens were collected via endotracheal suctioning.

All specimens were transported to the microbiology laboratory within two hours of collection. Microorganisms were identified primarily through conventional microbiological culture combined with antimicrobial susceptibility testing. In selected cases, especially when culture results were negative or incomplete, metagenomic next-generation sequencing and resistance gene detection were used to enhance pathogen identification and characterize antimicrobial resistance profiles.

### 2.5 Infection source determination

Patients found to be infected with MDRO at the time of admission or with MDRO detected upon entry were classified as having community-acquired infections or as being transferred from another department. Appropriate infection control measures were implemented accordingly.

### 2.6 Outcome assessment

#### 2.6.1 Primary outcomes

1. MDRO discovery rate: The MDRO discovery rate, defined as the proportion of patients identified with colonization or infection by MDROs through clinical cultures obtained during hospitalization, was compared between the intervention and control groups over the study period.
2. MDRO infection rate: The MDRO infection rate was compared between the intervention and control groups to assess the effectiveness of enhanced cleaning and disinfection measures in reducing MDRO infections.
3. Hospital-acquired infection rate: The overall hospital-acquired infection rate in the ICU was monitored and recorded for both groups, aiming to evaluate the impact of the intervention on controlling hospital infections.

#### 2.6.2 Secondary outcomes

1. ICU length of stay: The average ICU length of stay for both groups was compared to assess whether the intervention affected the duration of hospitalization.
2. Mechanical ventilation duration: The duration of mechanical ventilation was compared between the groups to evaluate whether the intervention had an impact on ventilator use.
3. Proportion of patients on mechanical ventilation: The proportion of patients receiving mechanical ventilation was compared to assess the intervention’s effect on ventilator support levels.
4. In-hospital mortality: In-hospital mortality rates were compared between the two groups to evaluate the clinical effectiveness of the intervention.
5. Total hospitalization days: Total hospitalization days were compared to determine if the intervention influenced overall hospitalization length.
6. Surface disinfection compliance rate: The surface disinfection compliance rate was defined as the percentage of surfaces with bacterial counts ≤ 5 CFU/cm² (General Administration of Quality Supervision, 2012; Ministry of Health of the People’s Republic of China, 2012). The compliance rate was calculated as (number of compliant tests/total number of tests) × 100.

### 2.7 Statistical methods

Data were analyzed using SPSS version 25.0 software. Continuous variables were expressed as means ± standard deviation and compared using t-tests. Categorical variables were expressed as percentages and compared using χ² tests. Multivariable logistic regression analysis was performed to identify factors independently associated with the outcome variables. A P-value < 0.05 was considered statistically significant. All data visualizations were generated using R version 4.4.3.

## 3 Results

### 3.1 Baseline characteristics

**Figure 1:**
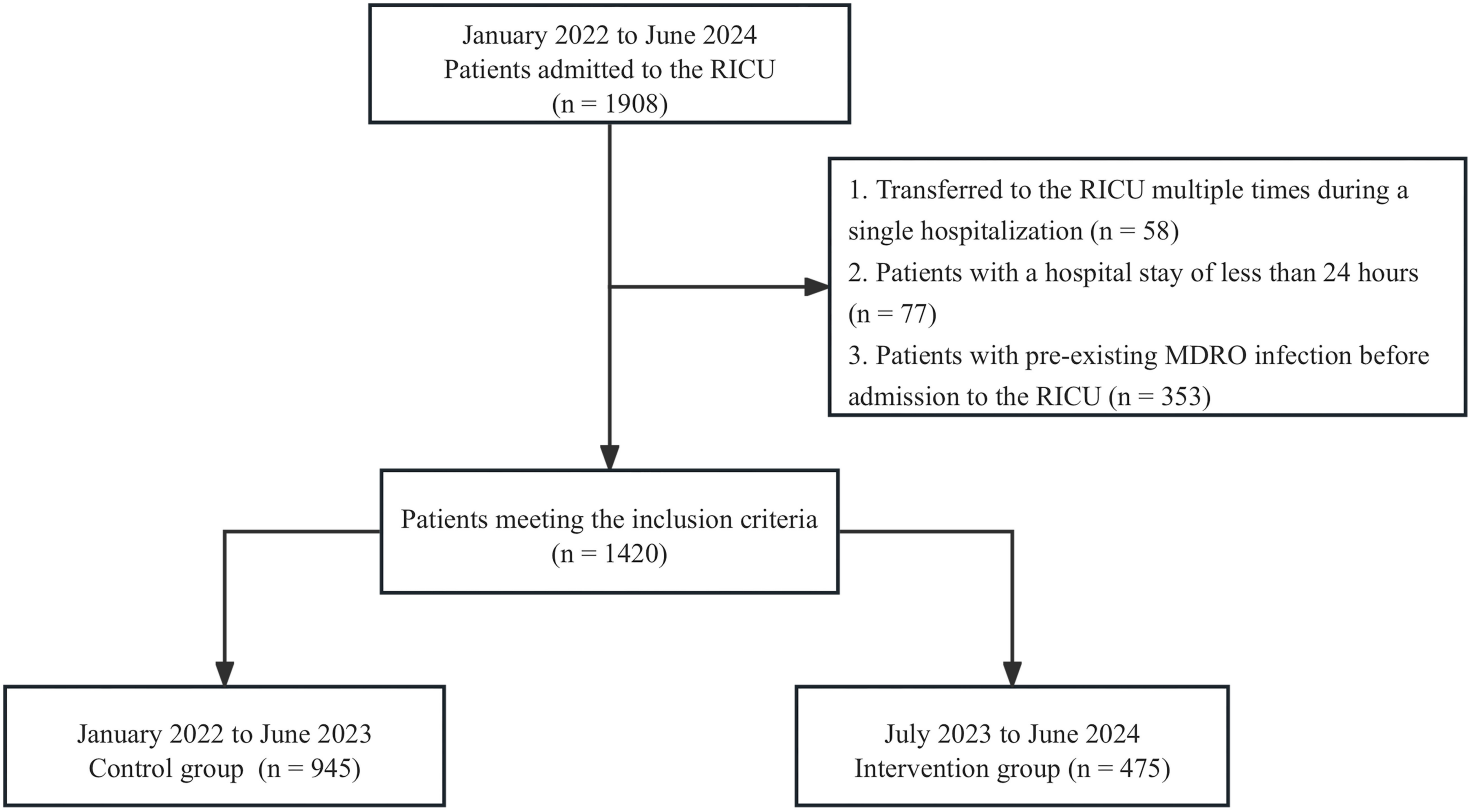
Flowchart.

A total of 1,908 patients were admitted to the Respiratory Intensive Care Unit (RICU) from January 2022 to June 2024, of which 1,420 met the inclusion criteria (**Figure 1**). The control group consisted of 945 patients with an average age of 69.81 ± 14.57 years, of which 653 (73.3%) were male. The intervention group included 475 patients with an average age of 70.42 ± 15.78 years, with 263 (55.4%) male patients. See **Table 1** and Flowchart 1. No significant differences were observed between the two groups in terms of age, comorbidities (except for chronic respiratory diseases), suspected infection sites, and disease severity assessments. However, the proportion of male patients in the control group was significantly higher than in the intervention group (69.1% vs. 55.4%, P < 0.001).

**Table 1:** Demographics and clinical characteristics of the study cohort.

Characteristics	Total (n=1420)	Intervention group (n=475)	Control group (n=945)	*P* values
Age, years	70.01 ± 14.98	69.81 ± 14.57	70.42 ± 15.78	0.473
Male (n, %)	916 (64.5)	653 (69.1)	263 (55.4)	**<0.001**
Commodity				
Chronic Respiratory Diseases (n, %)	515 (36.3)	312 (33.0)	203 (42.7)	**0.005**
Chronic Cardiovascular Diseases (n, %)	564 (39.7)	385 (40.7)	179 (37.7)	0.405
Malignant Tumors (n, %)	409 (28.8)	273 (28.9)	136 (28.6)	0.943
Chronic Digestive Diseases (n, %)	12 (0.8)	10 (1.1)	2 (0.4)	0.486
Cerebrovascular Diseases (n, %)	832 (58.6)	553 (58.5)	279 (58.8)	0.923
Suspected Infection Sites Upon ICU Admission				
Pulmonary (n, %)	998 (70.3)	666 (70.48)	332 (69.89)	0.821
Abdominal (n, %)	69 (4.9)	45 (4.76)	24 (5.05)	0.810
Blood (n, %)	269 (18.9)	278 (29.42)	151 (31.79)	0.358
Other Sites (n, %)	16 (1.1)	8 (0.89)	8 (1.68)	0.158
Disease Severity Score at Admission				
APACHE II Score	21.62 ± 9.00	21.24 ± 8.94	22.00 ± 9.06	0.434
SOFA Score	7.77 ± 4.54	7.79 ± 4.31	7.72 ± 4.96	0.385

APACHE II Score, Acute Physiology and Chronic Health Evaluation II Score; SOFA Score, Sequential Organ Failure Assessment Score.

Bold P values indicate P < 0.05.

### 3.2 Primary outcomes

The primary outcomes assessed were the MDRO discovery rate, MDRO infection rate, and hospital-acquired infection rate. The intervention group had a significantly lower MDRO discovery rate compared to the control group (35.2% vs. 45.9%, P < 0.001), indicating the effectiveness of the combined disinfection intervention in reducing the prevalence of MDROs in the ICU. The intervention group also showed a significantly lower MDRO infection rate (0.8% vs. 3.5%, P = 0.003). A detailed comparison of the MDRO species detected in the intervention and control groups is shown in **Supplementary Figure 1**. This suggests that the implementation of enhanced cleaning and disinfection practices significantly reduced the incidence of infections caused by MDROs. The overall hospital-acquired infection rate was significantly lower in the intervention group (1.5% vs. 3.5%, P = 0.030). This finding further supports the effectiveness of the intervention in reducing the occurrence of hospital-acquired infections in the ICU setting (**Table 2**).

**Table 2:** Comparison of primary outcomes between the intervention and control groups.

Characteristics	Total (n=1420)	Intervention group (n=475)	Control group (n=945)	*P* values
MDRO discovery rate (n, %)	601 (42.3)	167 (35.2)	434 (45.9)	**<0.001**
MDRO Infection Rate (n, %)	37 (2.6)	4 (0.8)	33 (3.5)	**0.003**
Hospital-Acquired Infection Rate (n, %)	40 (2.8)	7 (1.5)	33 (3.5)	**0.030**

MDRO, Multi-drug resistant organism.

Bold P values indicate P < 0.05.

### 3.3 Secondary outcomes

Several secondary outcomes were also assessed to evaluate the broader impact of the intervention on patient care. The average ICU length of stay was significantly longer in the intervention group (9.38 ± 11.99 days vs. 8.02 ± 11.50 days, P = 0.021), which may reflect the presence of more severe or prolonged cases requiring extended care. No significant difference was observed in the duration of mechanical ventilation between the two groups (14.74 ± 16.97 hours vs. 12.59 ± 20.66 hours, P = 0.131), indicating that the intervention did not notably affect the length of mechanical ventilation. However, the proportion of patients requiring mechanical ventilation was significantly higher in the intervention group (62.7% vs. 53.5%, P = 0.023), suggesting that patients in this group experienced more severe respiratory distress (**Table 3**).

The in-hospital mortality rate was significantly lower in the intervention group (13.5% vs. 19.5%, P = 0.005), highlighting the potential life-saving benefits of the intervention in critically ill patients. No significant difference was found in the total length of hospitalization between the two groups (9.99 ± 12.57 vs. 9.15 ± 14.08, P = 0.393), indicating that the intervention did not substantially affect overall hospital stay duration (**Table 3**).

**Table 3:** Comparison of secondary outcomes between the intervention and control groups.

Characteristics	Total (n=1420)	Intervention group (n=475)	Control group (n=945)	*P* values
In-Hospital Mortality (n, %)	248 (17.5)	64 (13.5)	184 (19.5)	**0.005**
Average Length of Hospital Stay, days	24.36 ± 26.29	23.14 + 18.98	24.81 ± 28.51	0.382
ICU Length of Stay, days	8.48 ± 11.68	9.38 ± 11.99	8.02 ± 11.50	**0.021**
Requiring Mechanical Ventilation (n, %)	804 (56.62)	298 (62.7)	506 (53.5)	**0.023**
Duration of Mechanical Ventilation, hours	13.39 ± 19.39	14.74 ± 16.97	12.59 ± 20.66	0.131
Costs, in 10k yuan	9.38 ± 13.69	9.99 ± 12.57	9.15 ± 14.08	0.393

MDRO, Multi-drug resistant organism.

Bold P values indicate P < 0.05.

The disinfection compliance rate was slightly higher in the intervention group (97.4% vs. 95.2%), though this difference was not statistically significant (P = 0.855). Nonetheless, this trend suggests that the intervention may have contributed to improved adherence to disinfection protocols.

## 4 Discussion

MDRO infections have become an increasingly serious public health issue, profoundly affecting patient health. According to the World Health Organization, antibiotic resistance is one of the greatest global health threats, particularly in ICUs, where the incidence of MDRO infections has significantly increased (Teng et al., 2023). These resistant bacteria not only prolong hospital stays but also significantly increase mortality rates, placing a considerable economic burden on healthcare systems. In this study, we retrospectively compared the effectiveness of strategies combining or not combining the unlimited use of disinfectant wipes with routine bundle interventions in preventing hospital-acquired infections and MDRO infections in RICU patients.

The emergence of MDROs is the result of multiple contributing factors. On one hand, the misuse or overuse of antibiotics is a significant driving force behind the development and spread of MDROs. Studies have shown that antibiotic selection pressure accelerates the acquisition and spread of resistance genes, particularly in healthcare settings, where the excessive use of broad-spectrum antibiotics significantly increases the risk of MDRO outbreaks (Kollef et al., 2021). Despite efforts to control antibiotic misuse through the promotion of rational antibiotic use and strict prescription oversight in recent years (Harris et al., 2016; He et al., 2019), the spread of MDROs has not been effectively contained. On the other hand, the transmission of colonizing bacteria on environmental surfaces may be another key pathway for the persistent prevalence of MDROs. Research indicates that MDROs can survive on hospital environmental surfaces (e.g., bed rails, doorknobs, medical equipment) for days or even weeks, spreading through contaminated medical devices and healthcare workers’ hands. This mode of transmission is covert and persistent, with higher colonization rates and greater transmission risks on high-touch surfaces. Studies have shown that in open ICUs with Acinetobacter baumannii infections, 9.5% of environmental surfaces were contaminated with resistant bacteria, compared to 13.4% in single-patient ICUs (Shimose et al., 2016). However, current supervision of environmental surface cleaning and disinfection remains insufficient. ICU patients, due to various invasive procedures, malnutrition, or immunosuppression, are at high risk for infections. Proper environmental cleaning and disinfection are critical in controlling hospital infections, particularly MDRO infections. Bundle interventions have been shown to reduce MDRO infection rates and hospital-acquired infection rates in ICU settings (Song et al., 2024; Wozniak et al., 2024). Additionally, disinfectant wipes are effective in killing MDROs (Song et al., 2019; Dadon et al., 2023).

Although previous studies have demonstrated that both bundle interventions and enhanced surface cleaning with disinfectant wipes can effectively reduce MDRO infection rates in hospitals, no research has explored the combined use of both, particularly with no restrictions on disinfectant wipe usage. Our study found that the intervention group (patients receiving routine bundle interventions combined with unlimited use of disinfectant wipes) had significantly lower MDRO discovery rates, hospital infection rates, and in-hospital mortality compared to the control group (patients receiving routine bundle interventions alone), highlighting the critical role of enhanced surface disinfection in controlling MDRO infections. This finding provides important evidence for the development of hospital infection control strategies.

Currently, commonly used disinfectants for high-touch surfaces in the ICU include quaternary ammonium salts, alcohols, iodophors, hydrogen peroxide, peracetic acid, chlorine dioxide, and chlorine-based disinfectants. According to the Guidelines of China, daily surface disinfection recommends using low-to-medium level disinfectants, with quaternary ammonium salts being the most commonly used (Ministry of Health and Family Planning of the People’s Republic of China, 2016). The disinfectant wipes used in our study contain a dual-chain quaternary ammonium salt disinfectant, which has excellent bactericidal activity and biofilm resistance with minimal corrosiveness (Zhi et al., 2022; Lv et al., 2024). Therefore, in the intervention group, we used these disinfectant wipes at least twice a day to clean high-touch surfaces around the patient’s environment, without limiting the quantity or frequency of use. Our intervention not only reduced MDRO discovery rates and hospital infection rates but also significantly lowered in-hospital mortality. Additionally, we found that the intervention group had a significantly higher average length of hospital stay and mechanical ventilation duration, which may be attributed to the older age, greater comorbidities, and more severe disease in these patients.

This study does have some limitations. Firstly, being a single-center retrospective study with a relatively small sample size, the generalizability of the results may be limited. Larger, multicenter, prospective studies are needed to enhance the reliability and applicability of the findings. Secondly, this study lacked certain evaluation metrics for the effectiveness of surface disinfection. Future studies should include these additional indicators. Thirdly, the intervention measures in this study did not address all potential factors affecting MDRO infections. Future research should consider more comprehensive intervention strategies, including the combined effects of different cleaning and disinfection methods, to improve the effectiveness of interventions. Lastly, this study did not directly assess the microbiological efficacy of disinfectant wipes in reducing environmental bioburden, as prior evidence has already demonstrated their effectiveness in lowering surface microbial loads.

In summary, our study demonstrates that the combination of disinfectant wipes with bundle management interventions effectively enhances cleaning and disinfection practices, leading to a significant reduction in MDRO infection rates and in-hospital mortality among ICU patients. These findings provide valuable empirical evidence to support infection control strategies in healthcare settings, highlighting the potential clinical value of this combined approach. While the study has certain limitations, the results offer important insights into improving MDRO infection management and underscore the critical importance of implementing effective cleaning and disinfection strategies within hospital environments.

## Data Availability

The original contributions presented in the study are included in the article/**Supplementary Material**. Further inquiries can be directed to the corresponding authors.

## Supplementary material

Supplementary Figure 1 Comparison of MDRO Detection and Infection Counts Between Intervention and Control Groups. **(A)** Comparison of multidrug-resistant organism (MDRO) detection counts between the intervention and control groups. Each bar represents the number of detections per bacterial species, stratified by group. **(B)** Comparison of infection counts caused by MDROs between the two groups. The height of each bar represents the total number of infections attributed to specific MDRO species.
