# Supplementary figures and images for: Impact of free provision of disinfectant wipes combined with bundle management on the prevention of multi-drug resistant organism infections in the respiratory and intensive care unit
Source: Front Cell Infect Microbiol. 2025 Jul 8;15:1581545. doi: 10.3389/fcimb.2025.1581545 (PMC12279828; doi:10.3389/fcimb.2025.1581545)

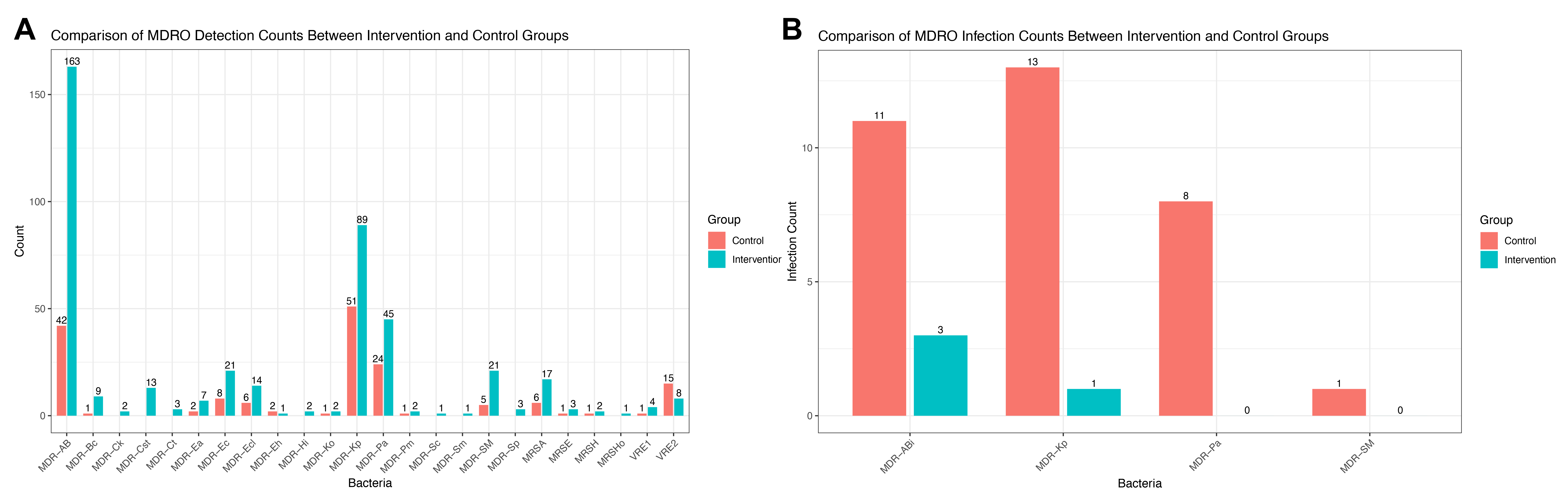

Supplement: Supplementary Figure 1 — Comparison of MDRO Detection and Infection Counts Between Intervention and Control Groups. (A) Comparison of multidrug-resistant organism (MDRO) detection counts between the intervention and control groups. Each bar represents the number of detections per bacterial species, stratified by group. (B) Comparison of infection counts caused by MDROs between the two groups. The height of each bar represents the total number of infections attributed to specific MDRO species. [file Image1.tif]
