# Supplementary material for: Impact of free provision of disinfectant wipes combined with bundle management on the prevention of multi-drug resistant organism infections in the respiratory and intensive care unit
Source: Front Cell Infect Microbiol. 2025 Jul 8;15:1581545. doi: 10.3389/fcimb.2025.1581545 (PMC12279828; doi:10.3389/fcimb.2025.1581545)
Supplement: Supplementary file 2 [file Table1.docx]

Supplementary Table 1 Effect of the Intervention on Clinical Outcomes in Unadjusted and Adjusted Logistic Regression Models

| **Models** | **Outcome** | **OR (95%CI)** | ***P* value** |
| --- | --- | --- | --- |
| Unadjusted model | MDRO discovery | 0.64 (0.51-0.80) | **<0.001** |
| Model 1 | MDRO discovery | 0.63 (0.50-0.79) | **<0.001** |
| Model 2 | MDRO discovery | 0.57 (0.45-0.72) | **<0.001** |
| Unadjusted model | MDRO infection | 0.23 (0.07-0.59) | **0.007** |
| Model 1 | MDRO infection | 0.23 (0.07-0.59) | **0.007** |
| Model 2 | MDRO infection | 0.22 (0.07-0.56) | **0.004** |
| Unadjusted model | Hospital-Acquired Infection | 0.41 (0.17-0.89) | **0.035** |
| Model 1 | Hospital-Acquired Infection | 0.41 (0.16-0.88) | **0.035** |
| Model 2 | Hospital-Acquired Infection | 0.39 (0.16-0.83) | **0.024** |
| Unadjusted model | In-Hospital Mortality | 0.64 (0.47-0.87) | **0.005** |
| Model 1 | In-Hospital Mortality | 0.64 (0.47-0.87) | **0.005** |
| Model 2 | In-Hospital Mortality | 0.53 (0.38-0.73) | **<0.001** |

Model 1: adjusted for age and sex

Model 2: adjusted for age, sex, chronic respiratory diseases, and mechanical ventilation.

CI：Confidence Interval; MDRO: Multidrug-Resistant Organism; OR：Odds Ratio.
